# Hepatocyte-Specific Ablation of PP2A Catalytic Subunit *α* Attenuates Liver Fibrosis Progression via TGF-*β*1/Smad Signaling

**DOI:** 10.1155/2015/794862

**Published:** 2015-02-01

**Authors:** Na Lu, Yun Liu, An Tang, Lulu Chen, Dengshun Miao, Xiaoqin Yuan

**Affiliations:** ^1^Department of Anatomy, Histology and Embryology, Nanjing Medical University, Nanjing, Jiangsu 210029, China; ^2^Model Animal Research Center of Nanjing University, Nanjing, Jiangsu 210061, China

## Abstract

Protein phosphatase 2A (PP2A), a family of the major serine/threonine phosphatases in cells, regulates many aspects of physiological processes. However, isoform-specific substrates and the biological role of each specific member of the PP2A family remain largely unknown. In this study, we investigated whether PP2A catalytic subunit C*α* (PP2Ac*α*) is involved in chronic hepatic injury and fibrosis. A hepatocyte-specific PP2Ac*α* ablation mice model was established to examine the effect of PP2Ac*α* on carbon tetrachloride- (CCl4-) induced chronic hepatic injury and fibrosis. Our results showed that PP2Ac*α* knockout mice were less susceptible to chronic CCl4-induced liver injury as evidenced by lower levels of serum alanine aminotransferase and aspartate aminotransferase, decreased hepatocyte proliferation, and increased rate of apoptotic removal of the injured hepatocytes. PP2Ac*α* knockout mice also displayed a lesser extent of liver fibrosis as a significant decrease in the proportion of *α*-smooth muscle actin-expressing cells and collagen deposition was observed in their liver tissues. Furthermore, the levels of serum TGF-*β*1 and hepatocytic Smad phosphorylation were reduced in the PP2Ac*α* knockout mice. These data suggest that hepatocyte-specific ablation of PP2Ac*α* protects against CCl4-induced chronic hepatic injury and fibrogenesis and the protective effect is mediated at least partially through the impaired TGF-*β*1/Smad signaling.

## 1. Introduction

Hepatic fibrosis is a common pathological consequence of chronic liver diseases and usually results from prolonged liver injury caused by chronic hepatitis, alcohol, or chemical insults [1]. Irrespective of the etiology, persisting liver fibrogenesis is widely recognized as the major driving force for the progression of any form of chronic liver disease (CLD) ultimately leading to liver cirrhosis and hepatic failure [2–5]. Among these lines, cirrhosis is currently defined as the end stage of hepatic fibrosis with high prevalence in the world and closely associated with hepatocellular carcinoma incidence. Thus, extensive efforts have been made at elucidating hepatic fibrogenesis with the aim of developing therapeutic strategies for abrogating its progression [6, 7].

Hepatic fibrosis is an abnormal response of the liver to persistent injury with an excessive and aberrant deposition of extracellular matrix (ECM) proteins in the liver, the most abundant of which is the collagen family [8]. After an injury, parenchymal cells undergo the process of regeneration and replacement of the necrotic or apoptotic cells, which requires both a well-orchestrated proliferation of cells and the reconstruction of ECM. When the injury persists, the damaged tissues would eventually suffer from extensive pathological fibrosis due to a progressive excess accumulation of ECM components in an attempt to limit the consequences of chronic parenchymal injury. In the past decade, although several novel mediators, mechanisms, and signaling pathways have been proposed to play an active role in sustaining liver fibrogenesis during CLD progression, transforming growth factor-*β*1 (TGF-*β*1) is considered as a major profibrogenic cytokine and a potent inducer of hepatic stellate cell (HSC) proliferation and collagen production [9]. Its collagen synthesis stimulatory effect is executed through intracellular signal transducers Smads whose phosphorylation and subsequent translocation into the nucleus upon TGF-*β*1-induced activation of the TGF-*β* receptor would regulate expression of profibrotic target genes [10].

Protein phosphatase 2A (PP2A) is the major eukaryotic serine/threonine phosphatase representing 0.1–1% of total cellular proteins and plays a crucial role in regulating most cellular functions [11]. The typical and primary mammalian PP2A is a heterotrimeric complex consisting of a scaffold subunit (A subunit), a catalytic subunit (PP2Ac), and a regulatory subunit (B subunit) [12]. Molecular cloning has disclosed that there are two isoforms of the mammalian PP2Ac: PP2Ac*α* (encoded by the* Ppp2ca* gene) and PP2Ac*β* (encoded by the* Ppp2cb* gene). These two isoforms share 97% homology in the amino acid sequence and the difference is within the first 30 amino acids [13]. Both PP2Ac isoforms are ubiquitously expressed with PP2Ac*α* transcripts 10-fold greater in general than PP2Ac*β* transcripts due to transcriptional regulation [14, 15]. However, despite the tremendous functional significance of the “PP2A” family, isoform-specific substrates and the biological role of each specific member of the “PP2A” family remain largely unknown because of the lack of valid isoform-specific PP2A antibodies or specific inhibitors/activators and activity assays, as well as the complexity of PP2A regulation. PP2A has been implicated in the TGF-*β*1 signaling pathway by modulating the basal level and activity of type I receptors that specifically phosphorylates Smad2 and Smad3 [16–18]. We have previously shown that conditionally inactivated* Ppp2ca* gene in hematopoietic cells perturbed fetal liver erythropoiesis and increased apoptosis of committed erythroid cells via the STAT5 pathway [19]. In this study, we investigated whether and how PP2Ac*α* was involved in hepatic fibrosis chronically induced by CCl_4_ using a genetic PP2Ac*α* ablation mice model. We found that PP2Ac*α* knockout mice were protected against liver injury and fibrosis development as compared with PP2Ac*α* wild-type mice, an effect likely attributed to a defect in TGF-*β*1/Smad signaling.

## 2. Materials and Methods

### 2.1. Mice and Treatment

*Ppp2ca* ^*fl/fl*^ mice and wild-type B6 mice were bred with* AlbCre* ^*+*^ mice, respectively. All the mice were of a mixed 129/B6 background. After cross mating,* Ppp2ca* ^*fl/fl*^ */AlbCre* ^*+*^ (i.e., knockout, KO),* Ppp2ca* ^*fl/fl*^ and* AlbCre* ^*+*^ were generated and used in the experiments. Since there was no significant difference in PP2Ac isoform expression, enzyme activity, and liver fibrosis phenotype between* Ppp2ca* ^*fl/fl*^ mice and* AlbCre* ^*+*^ mice, these two genotypic mice were used as control. Animal welfare and experimental procedures were approved by the Institutional Animal Care and Use Committee of Nanjing Medical University. To establish chronic liver fibrosis, male mice aged 8–10 weeks were intraperitoneally injected with 2 mg/kg body weight of 10% CCl_4_ dissolved in olive oil 3 times a week for 5 weeks. Mice were euthanized 48 h following the last injection. Before mice were sacrificed, serum was obtained by retroorbital bleeding from anesthetized mice following overnight fasting.

### 2.2. Measurement of Phosphatase Activity

Liver protein was extracted in a phosphatase extraction buffer containing 20 mmol/L imidazole-HCl, 2 mmol/L EDTA, 2 mmol/L EGTA (pH 7.0), 1 mmol/L benzamidine, 1 mmol/L phenylmethylsulfonyl fluoride, and protein inhibitor cocktails. Phosphatase activity was assayed using a malachite green-based PP2A Assay Kit (Upstate Biotechnology, Waltham, MA). Briefly, total proteins were immunoprecipitated with anti-PP2Ac, and PP2Ac-bound beads were incubated with synthetic phosphopeptide for the dephosphorylation reaction. The reaction supernatant was then mixed with malachite green reagent for color development. Changes in absorbance were measured at 650 nm.

### 2.3. ELISA Assay and Biochemistry Analysis

Mice serum TGF-*β*1 level was assayed by enzyme-linked immunosorbent assay (ELISA) with mouse TGF-*β*1 immunoassay kit (Sunny ELISA Kits, Mutisciences, Hangzhou, China) following manufacturer's instructions. Serum alanine transaminase (ALT) and aspartate aminotransferase (AST) levels were measured using an autoanalyzer.

### 2.4. Immunohistochemistry

Immunohistochemisty was performed on paraffin embedded liver tissues using antibodies specific to PP2Ac (Abcam, Cambridge, UK), *α*-SMA (Abcam, Cambridge, UK), and PCNA (Abcam, Cambridge, UK). Dewaxed and rehydrated paraffin-embedded sections were incubated with methanol: hydrogen peroxide (1 : 10) to block endogenous peroxidase activity and then were washed in Tris-buffered saline (pH 7.6). The slides were then incubated with the primary antibodies overnight at room temperature. After rinsing with Tris-buffered saline for 15 min, tissues were incubated with secondary antibody (biotinylated goat anti-rabbit IgG, Sigma). Sections were then washed and incubated with the Vectastain Elite ABC reagent (Vector Laboratories, Burlingame, CA) for 45 min. Staining was developed using 3,3-diaminobenzidine (2.5 mg/mL) followed by counterstaining with Mayer's hematoxylin. Five high-power fields (400x magnification) were randomly selected within each slide. Data are expressed as the average percentage of positive staining cells.

### 2.5. Histological Analysis and Collagen Content Measurement

Liver tissues were fixed in 4% formalin and embedded in paraffin according to standard procedure. 5 *μ*m sections were cut on a rotary microtome and stained with Sirius red and trichrome for collagen content measurement. For quantification of collagen deposition, slides were prepared from the tissues of 5 individual mice of each genotype. Five fields were randomly selected per slide and calculated for collagen accumulation using Image Pro-Plus software under ×100 objective.

### 2.6. TUNEL Staining

For* in situ* detection of apoptotic cells, terminal deoxynucleotidyl transferase-mediated labeling of nick-end DNA (TUNEL) staining was performed according to the manufacturer's instructions (Roche). Five high-power fields were randomly selected per slide at 400x magnification. Data are expressed as the average percentage of TUNEL-positive cells.

### 2.7. Western Blot Analysis

Lysates from liver tissues were separated on SDS-PAGE, transferred to polyvinylidene fluoride (PVDF) membranes, and blotted with primary antibodies directed against PP2Ac (Abcam, Cambridge, UK), PCNA (Abcam, Cambridge, UK), Bax (Cell Signaling Technology, Danvers, MA), cleaved caspase-3 (Abcam, Cambridge, UK), *α*-smooth muscle actin (*α*-SMA, Abcam, Cambridge, UK), Smad2, phospho-Smad2, Smad3, phospho-Smad3, Bcl-2, *β*-actin (all from Cell Signaling Technology, Danvers, MA), Cyclin D1 (Upstate Biotechnology, Lake Placid, NY), and Cyclin E (Merck Millipore, Billerica, MA), followed by appropriate secondary antibodies and chemiluminescent detection.

### 2.8. Statistical Analysis

All data are presented as means ± standard deviation (SD). Student's *t*-test was used for comparisons. *P* < 0.05 was considered statistically significant.

## 3. Results

### 3.1. PP2Ac*α* Knockout Mice Were Protected against CCl_4_-Induced Chronic Hepatic Injury

**Figure 1:**
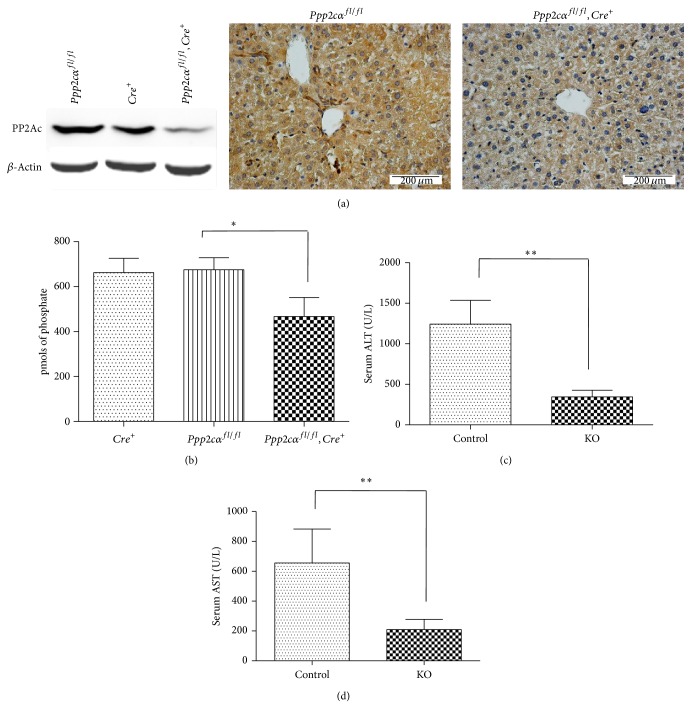
PP2Ac*α* knockout alleviated liver injury induced by chronic CCl_4_ administration. (a) PP2Ac protein levels in the livers of knockout (*Ppp2cα* ^*fl/fl*^ */AlbCre* ^*+*^) and control (*Ppp2cα* ^*fl/fl*^ and* AlbCre* ^*+*^) mice were determined by Western blot analysis (left panel) and immunohistochemical staining (right panel) using an antibody detecting both PP2Ac*α* and PP2Ac*β*. Magnification, ×400. Bar, 200 *μ*m. (b) PP2A phosphatase activity of protein extracts from the mice liver tissues. Each value is mean ± SD (*n* = 8), ^*^ *P* < 0.05. (c, d) Serum alanine aminotransferase (ALT) and aspartate aminotransferase (AST) levels in the knockout and control mice 48 h after intraperitoneal injection with 2 mg/kg CCl_4_ three times a week for 5 weeks. Each value is mean ± SD (*n* = 8), ^**^ *P* < 0.01.

To examine a deletion efficiency of PP2Ac*α* in hepatocytes of PP2Ac*α* knockout (*Ppp2cα* ^*fl/fl*^ */AlbCre* ^*+*^) mice, PP2Ac protein contents and PP2A phosphatase activity were measured from the livers of* Ppp2cα* ^*fl/fl*^ */AlbCre* ^*+*^ mice and control* Ppp2cα* ^*fl/fl*^ and* AlbCre* ^*+*^ mice. As shown in Figure 1(a), the expression level of PP2Ac was substantially reduced in* Ppp2cα* ^*fl/fl*^ */AlbCre* ^*+*^ mice livers compared to that in either* Ppp2cα* ^*fl/fl*^ or* AlbCre* ^*+*^ control, when measured by both Western blot analysis and immunochemical staining using an antibody detecting both PP2Ac*α* and PP2Ac*β*. Similarly, PP2A phosphatase activity was significantly decreased in the livers of the PP2Ac*α* knockout mice (Figure 1(b). Since* Ppp2cα* ^*fl/fl*^ and* AlbCre* ^*+*^ mice displayed similar level of PP2Ac protein and phosphatase activity in livers, we used* Ppp2cα* ^*fl/fl*^ mice for subsequent studies. To determine whether PP2Ac*α* knockout mice could be protected against chronic liver injury, the mice were subjected to CCl_4_ treatment three times a week for 5 weeks and the two indicators of acute liver injuries, serum ALT and AST, were measured.  Figure 1(c) shows that the levels of serum ALT and AST in the knockout mice were significantly lower compared with the control, suggesting that PP2Ac*α* knockout mice were more resistant to CCl_4_-induced chronic hepatic damage.

### 3.2. Knockout of PP2Ac*α* Alleviated CCl_4_-Induced Chronic Hepatic Fibrosis

**Figure 2:**
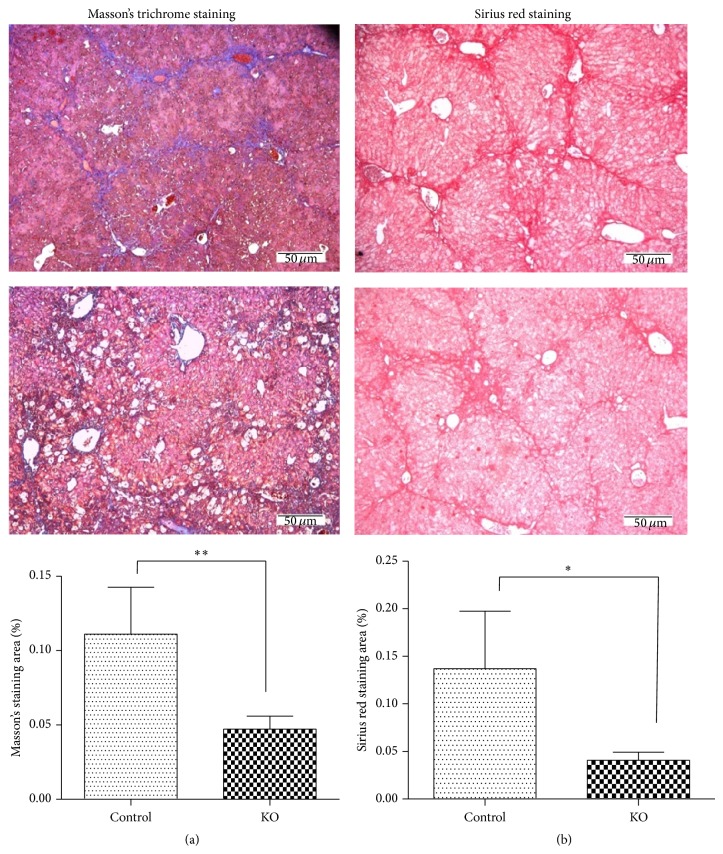
PP2Ac*α* ablation attenuated CCl_4_-induced liver fibrosis. Masson's trichrome staining (a) and Sirius red staining (b) of liver tissue sections from the control and PP2Ac*α* knockout mice at 5 weeks after the chronic CCl_4_ treatment. Magnification, ×100. Bar, 50 *μ*m. The histograms show the mean percentage of the staining area per total liver area determined from five randomly selected fields. Values are means ± SD, *n* = 5. ^*^ *P* < 0.05 and ^**^ *P* < 0.01.

To assess the potential protective effect of PP2Ac*α* deletion on liver fibrosis, collagen deposition was determined in the liver tissues of the control and PP2Ac*α* knockout mice after the chronic CCl_4_ administration.  Figure 2(a) shows representative micrographs of Masson's trichrome staining of liver tissue sections 5 weeks after the CCl_4_ treatment. In the liver tissues of control mice, extensive fibrosis displaying a honeycomb pattern of fibrous septa (blue staining) was evident whereas much weaker collagen staining was found in the livers of PP2Ac*α* knockout mice. The percentage of the fibrotic area per total liver area was reduced by 56% in the knockout mice when compared with the control mice. Consistently with the results obtained from Masson's trichrome staining, Sirius red staining for collagen deposition also showed that PP2Ac*α* knockout mice largely inhibited hepatic collagen accumulation after the chronic CCl_4_ challenge (Figure 2(b). These data indicated that loss of PP2Ac*α* was capable of protecting the liver from the development of fibrotic lesions after CCl_4_.

### 3.3. PP2Ac*α* Deletion Blocked Hepatic Stellate Cell (HSC) Activation* In Vivo*

**Figure 3:**
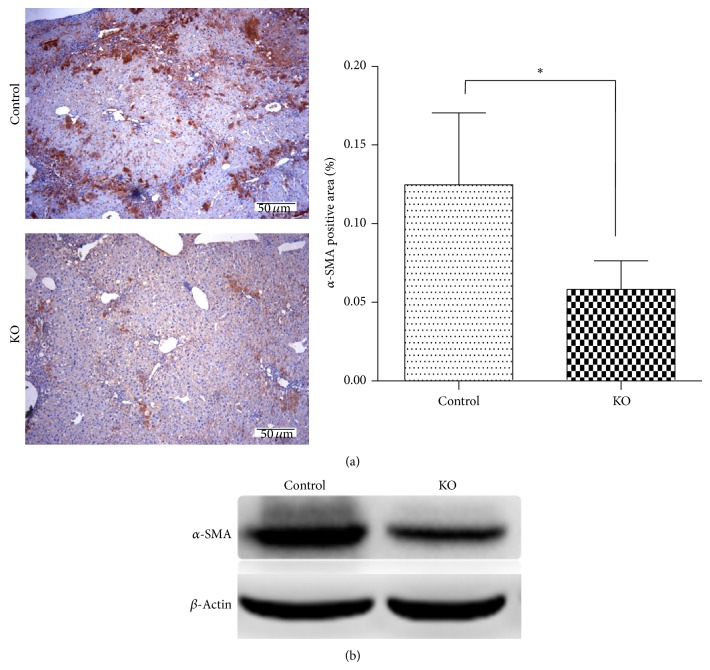
PP2Ac*α* deletion inhibited HSC activation. (a) Immunohistochemical analysis of *α*-SMA expression in liver tissues from the control and PP2Ac*α* knockout mice at 5 weeks after the chronic CCl_4_ treatment. Magnification, ×100. Bar, 50 *μ*m. The histogram shows the mean percentage of the *α*-SMA positive area per total liver area determined from five randomly selected fields. Values are means ± SD, *n* = 5. ^*^ *P* < 0.05. (b) Western blot analyses of hepatic *α*-SMA expression in the control and PP2Ac*α* knockout mice at 5 weeks after the chronic CCl_4_ treatment. Liver homogenates were probed with antibodies against *α*-SMA and *β*-actin, respectively.

Since activation of HSCs to overproduce ECM is a key event in the pathophysiology of hepatic fibrosis and *α*-smooth muscle actin (*α*-SMA) is a marker for activated HSCs, we sought to investigate whether there was different expression of *α*-SMA expression in livers from the control and PP2Ac*α* knockout mice chronically treated with CCl_4_. As shown in Figure 3(a), stronger intensity of *α*-SMA immunohistochemical staining and significantly more *α*-SMA positive cells were observed in liver sections of the control mice than those of PP2Ac*α* mice. Similar results were obtained from immunoblot analysis. Compared with the control, the levels of hepatic *α*-SMA protein were markedly reduced in the PP2Ac*α* knockout mice (Figure 3(b). These observations imply that PP2Ac*α* may stimulate liver fibrosis via HSC activation and therefore its deletion may reduce the liver fibrosis through abolishing the profibrogenic effect of CCl_4_.

### 3.4. Deletion of PP2Ac*α* Reduced Hepatocyte Proliferation but Increased Hepatocyte Apoptosis

**Figure 4:**
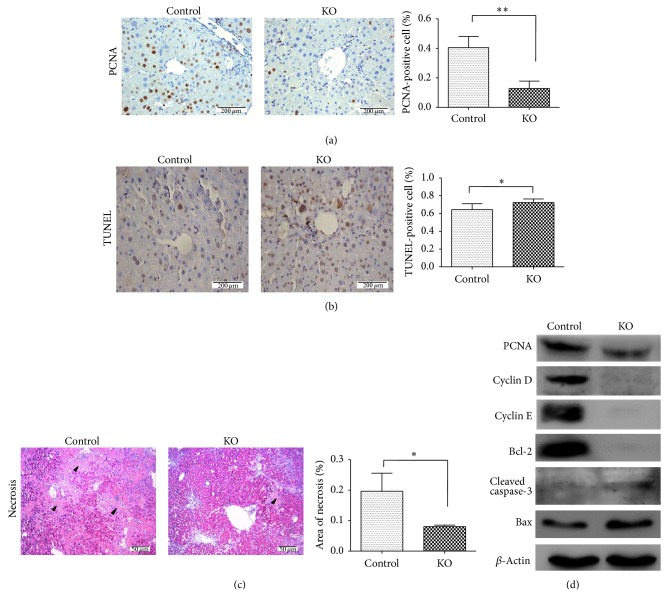
Knockout of PP2Ac*α* suppressed hepatocyte proliferation and enhanced hepatocyte apoptosis. (a) Immunohistochemical analysis of PCNA expression in the livers from the control and PP2Ac*α* knockout mice at 5 weeks after the chronic CCl_4_ treatment. Magnification, ×400. Bar, 200 *μ*m. The histogram shows the mean percentage of the PCNA-positive cells determined from five randomly selected fields. (b) TUNEL staining was performed on the liver sections from the control and PP2Ac*α* knockout mice at 5 weeks after the chronic CCl_4_ treatment. Magnification, ×400. Bar, 200 *μ*m. The histogram shows the mean percentage of the TUNEL-positive cells determined from five randomly selected fields. (c) H&E staining of liver tissue sections from the control and PP2Ac*α* knockout mice at 5 weeks after the chronic CCl_4_ treatment. Representative areas of necrosis are indicated by arrowheads. Magnification, ×100. Bar, 50 *μ*m. The histogram shows the mean percentage of the necrotic area per total liver area determined from five randomly selected fields. Values are means ± SD, *n* = 5. ^*^ *P* < 0.05 and ^**^ *P* < 0.01. (d) Western blot analyses of hepatic PCNA, Cyclin D, Cyclin E, Bcl-2, Bax, and cleaved caspase-3 expression in the control and PP2Ac*α* knockout mice at 5 weeks after the chronic CCl_4_ treatment. *β*-actin was used as a loading control.

To understand the mechanism underlying the less liver fibrosis in the PP2Ac*α* knockout mice than in the control mice, we first examined hepatocyte proliferation and apoptosis in the two strains of mice after the chronic challenge. As shown in Figure 4(a), hepatocyte proliferation was significantly reduced in the knockout mice compared to the control, as evidenced by PCNA immunohistochemical staining on liver sections. To determine whether the proliferation inhibitory effect induced by PP2Ac*α* knockout was related to apoptosis, we assessed the frequency of apoptotic cells within the liver tissues by the* in situ* TUNEL staining. As shown in Figure 4(b), a marginal increase in the number of apoptotic hepatocytes was observed in the PP2Ac*α* knockout mice compared to the control mice. However, necrosis was less pronounced in the liver of PP2Ac*α* knockout mice (Figure 4(c). Consistently with the apoptosis data, the liver lysates from PP2Ac*α* knockout mice showed a decrease in proliferating cell nuclear antigen PCNA, cell cycle-promoting molecule Cyclins D and E, and antiapoptotic molecule Bcl-2 expression levels but demonstrated an increased level of proapoptotic protein Bax and active caspase-3 (Figure 4(d)). These data indicated that the PP2Ac*α* knockout mice were protected against chronic CCl_4_-induced liver injury, possibly due to less injurious responsiveness in the lack of PP2Ac*α*.

### 3.5. PP2Ac*α* Deletion Inhibited TGF-*β* Secretion and Impaired TGF-*β* Downstream Signaling

**Figure 5:**
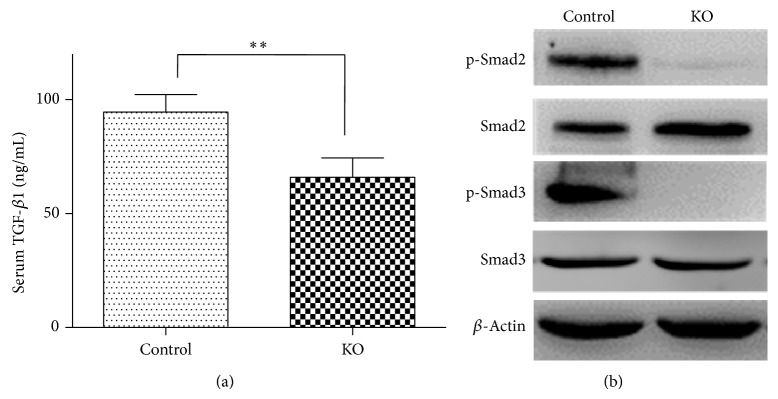
PP2Ac*α* ablation diminished the serum level of TGF-*β*1 and its downstream signal transduction. (a) Quantification of serum TGF-*β*1 level by ELISA in the control and PP2Ac*α* knockout mice chronically treated with CCl_4_ for 5 weeks. Values are means ± SD, *n* = 8. ^**^ *P* < 0.01. (b) Western blot analysis of phospho-Smad2 (p-Smad2), total Smad2 (Smad2), phospho-Smad3 (p-Smad3), and total Smad3 (Smad3) expression levels in the livers from the control and PP2Ac*α* knockout mice treated chronically with CCl_4_ for 5 weeks.

Since TGF-*β* and its downstream signal transducers Smads have been well established to play a vital role in the progression of fibrogenesis at both cellular and molecular levels [20], we next sought to determine whether TGF*β*1/Smad signaling was altered in the PP2Ac*α* knockout mice that may underlie the inner mechanism behind its resistance to CCl_4_-induced fibrosis. As shown in Figure 5(a), the serum level of TGF*β*1 was significantly reduced in the PP2Ac*α* knockout mice compared with wild-type mice. Meanwhile, the expressions of phospho-Smad2 and phospho-Smad3, mediators of the TGF*β*1 signaling, were also substantially suppressed in the PP2Ac*α* knockout mice. Thus, at least part of the observed reduction in hepatic fibrosis in the PP2Ac*α* knockout mice can be accounted for by a decrease in TGF*β*1/Smad signaling.

## 4. Discussion

This study demonstrates that PP2Ac*α* is crucial for liver injury and fibrogenesis. Conditional genetic deletion of PP2Ac*α* attenuated liver fibrosis in the mice following chronic CCl_4_ treatment, probably through impairing TGF*β*1/Smad profibrotic signaling pathway. Our results indicate that PP2Ac*α* could interfere with the TGF-*β*/Smad signaling pathway, which in turn modulates critical pathological events such as collagen deposition, HSC activation, and hepatocyte proliferation and apoptosis in the development of hepatic fibrosis.

PP2A regulates many key cellular processes such as the cell cycle, cell growth, apoptosis, and signal transduction. It has long been speculated that the individual, yet highly homologous, members of each subfamily of PP2A subunits would have unique and specific roles in addition to their established redundant activities. Genetic deletion of PP2A catalytic subunit C*α* (PP2Ac*α*) in mice results in embryonic lethality with no mesoderm induction [21], which implies that PP2Ac*α* plays an important role in mesenchymal cells such as osteoblasts, adipocytes, and myoblasts. Most recently, PP2Ac*α* has been shown to be an important regulator of adipocyte differentiation by regulating the expression of adipocyte marker genes and the Wnt/GSK-3*β*/*β*-catenin pathway [22]. However, the role of PP2Ac*α* in liver injury and fibrosis has not been examined previously. The results of the studies reported here provide multiple lines of evidence that the expression of PP2Ac*α* influences the consequence of hepatic damage repair and fibrogenesis under CCl_4_-induced liver toxicity. When treated chronically with CCl_4_, PP2Ac*α* knockdown mice had less liver damage as demonstrated by lower serum ALT and AST levels consequent to a significant reduction in PP2A phosphatase activity of the mice liver tissues. Decreased mitotic activity in the liver sections was further confirmed by immunostaining for expression of PCNA, a nuclear protein highly expressed during the DNA synthesis phase of the cell cycle and closely correlated with the proliferative state of the cells [23]. The number of PCNA-positive cells was significantly reduced in the livers of CCl_4_-treated PP2Ac*α* knockdown mice compared with the wild-type mice. The primary mode of cell death in response to CCl_4_ injury is through necrosis; however, it has been shown that apoptosis may also play a role in the elimination of damaged hepatocytes [24–26]. In our mice model under chronic CCl_4_ administration, increased hepatocyte apoptosis and reduced antiapoptotic protein Bcl-2 expression as well as enhanced expression of proapoptotic protein Bax and activation of caspase-3 were observed in livers of PP2Ac*α* knockdown mice. It may be noteworthy that treatment of PP2A inhibitor could augment Bcl-2 phosphorylation that also leads to a reduction in its antiapoptotic function [27]. Therefore, reduced Bcl-2 expression together with possible increased Bcl-2 phosphorylation may contribute to higher apoptotic rate in the hepatocytes of PP2Ac*α* mice. These results suggest that the ablation of the hepatocyte-specific PP2Ac*α* reduces the severity of CCl_4_-induced liver injury by masking proliferative/regenerative response and promoting apoptotic response to remove the damaged hepatocytes.

Activation of the *α*-SMA-positive, matrix-producing myofibroblasts has long been recognized as a major decisive event in tissue fibrogenesis after chronic injury. They are often presumed to be derived from hepatic stellate cells (HSCs) which have been considered as the main source of ECM during liver fibrosis and are the first identified fibrogenic cell population [28, 29]. In the normal liver, they display a quiescent phenotype. However, upon acute or chronic liver injury, a complex network of autocrine/paracrine fibrogenic signals promotes transdifferentiation of quiescent HSCs to a myofibroblastic phenotype characterized by the expression of *α*-smooth muscle actin and de novo expression of receptors for fibrogenic, chemotactic, and mitogenic factors [30]. In this study, we have shown that significantly less collagen accumulation in the livers of PP2Ac*α* knockdown mice was accompanied by a substantial reduction in *α*-SMA-expressing cells. It is therefore possible that suppressed HSC activation due to PP2Ac*α* ablation may contribute to the resistance to chronic hepatic fibrosis in the PP2Ac*α* knockout mice compared to the similarly treated wild-type mice. HSCs are not only an important source of growth factors in the liver but also a good responder to these factors, emphasizing the importance of tightly regulated autocrine control of growth factor activity within pericellular milieu [31]. TGF-*β*1 is derived from both paracrine and autocrine sources and is the most potent fibrogenic cytokine in the liver [32, 33]. It is secreted in a biologically inactive form which can be converted into active form in response to injury. Once activated, TGF-*β*1 signals via its cognate TGF-*β*1 receptor that phosphorylates the transcription factor Smads (Smads 1, 2, 3, 5, and 8) forming a complex with the co-Smad (Smad4), which then translocates into the nucleus to regulate transcription of profibrotic target genes [20, 33]. TGF-*β*1/Smad signaling pathway can influence various aspects in the fibrogenic process including regulation of hepatocyte proliferation and apoptosis, mediation of HSC activation, and the subsequent ECM production in response to liver injury [34, 35]. In our CCl_4_ chronically treated mice models, the TGF-*β*1 serum level was significantly decreased in PP2Ac*α* knockout mice compared with the control wild-type mice. As expected, phospho-Smad2 and phospho-Smad3 were drastically reduced in the absence of PP2Ac*α*. In good agreement with this, we found a similar change in the expression of Cyclin D1 and Cyclin E which have been known to be TGF-*β*1/Smad regulated targets [36, 37]. PP2A has been reported to function as a negative regulator in TGF-*β*1-induced TAK1 (transforming growth factor-*β*-activated kinase 1) activation in mesangial cells [38]. However, it should be recognized that control of TAK1 activation may be conducted in a cell type-specific or context-dependent manner [38, 39]. Thus, it is conceivable that different protein phosphatases or even different subunits of PP2A may be compensatorily expressed due to PP2Ac*α* ablation and play distinct and opposing roles in the regulation of TGF-*β*1/Smad signaling. While the exact molecular mechanisms remain to be clarified and explored, it is tempting to suggest a model in which TGF-*β*1/Smad signaling is impaired as a result of PP2Ac*α* deficiency, which in turn leads to less susceptibility to hepatic damage, suppressed HSC activation, and decreased collagen production, thus protecting PP2Ac*α* knockout mice against CCl_4_-induced chronic hepatic fibrosis.

In summary, our studies demonstrate that PP2Ac*α* plays a crucial role in the progression of fibrosis using an* in vivo* genetic mice model. In PP2Ac*α* knockout mice, chronic hepatic fibrosis induced by CCl_4_ administration is less severe compared with their wild-type littermates. The protective effect of PP2Ac*α* ablation is mediated, at least in part, through the impaired TGF-*β*1/Smad signaling in PP2Ac*α* mice. Therefore, genetic approach or pharmacological intervention targeting PP2Ac*α* enzymatic activity or its interaction with downstream targets could potentially serve as an effective strategy to the future treatment of hepatic fibrosis.
